# Epidemiological characteristics of human parainfluenza virus infection in hospitalized children: a single-center study

**DOI:** 10.3389/fpubh.2026.1833325

**Published:** 2026-06-19

**Authors:** Yuzhe Guo, Huilin Zhou, Weiqin Jiang, Jing Wang, Yujuan Huang

**Affiliations:** Department of Emergency, Shanghai Children’s Hospital, School of Medicine, Shanghai Jiao Tong University, Shanghai, China

**Keywords:** acute respiratory tract infections, children, coinfections, epidemiology, human parainfluenza virus, seasonal variation

## Abstract

**Objective:**

This study aims to investigate the epidemiological characteristics of human parainfluenza virus (HPIV) infections in hospitalized children with acute respiratory tract infections (ARTIs) at a single center in Shanghai, and to provide scientific evidence for optimizing diagnostic and therapeutic strategies for pediatric ARTIs.

**Methods:**

A retrospective study was conducted on 29,260 children hospitalized with ARTIs at Shanghai Children’s Hospital between January 2021 and December 2024. The study analyzed the sex distribution, age-specific patterns, seasonal variations, and coinfection characteristics of HPIV infections.

**Results:**

Among the 29,260 children with ARTIs, the overall detection rate of HPIV was 5.1% (1,487/29,260). The highest positivity rates were observed in children aged <1 year (7.4%) and 1–3 years (8.1%), while the lowest was in those aged 13–18 years (1.7%; overall *p* < 0.001). No significant difference in detection rates was observed between male (5.3%, 845/16,056) and female patients (4.9%, 642/13,204) (*p* > 0.05). HPIV infections peaked in summer (9.4%), with detection rates significantly higher than in other seasons (all *p* < 0.001). The detection rate in 2021 (7.2%, 364/5,053) was significantly higher than in subsequent years (*p* < 0.001). Coinfections were detected in 28.9% (429/1,487) of HPIV-positive cases, with common co-pathogens including rhinovirus (35.2%, 151/429), *Mycoplasma pneumoniae* (14.5%, 62/429), and respiratory syncytial virus (13.3%, 57/429). Mixed infections were independently associated with a higher risk of severe pneumonia (adjusted odds ratio 2.45; *p* < 0.001).

**Conclusion:**

HPIV is a significant cause of hospitalization for ARTIs in children in Shanghai, especially among those under 3 years of age, with a distinct seasonal peak in summer. Mixed HPIV infections, particularly with rhinovirus and *Mycoplasma pneumoniae*, are associated with more severe disease. These findings support the need for heightened clinical awareness, targeted testing during peak seasons, and consideration of mixed infection status in patient management strategies.

## 1 Introduction

Human parainfluenza virus (HPIV), a significant pathogen of pediatric respiratory infections worldwide, is associated with a substantial disease burden (1, 2). It accounts for approximately 6–15% of global pediatric respiratory infection hospitalizations (3–5). While infections in immunocompromised individuals can lead to severe outcomes (6, 7), HPIV poses a persistent threat to all young children due to its characteristic of causing recurrent infections (8, 9).

In the later stages of the COVID-19 pandemic, with the phased adjustment of nonpharmaceutical interventions (NPIs), the epidemiological pattern of HPIV has undergone significant changes. For example, after Finland lifted restrictions, the detection rate of HPIV in children experienced an abnormal autumn surge (10). Similar phenomena of altered seasonality have also been reported in South China (11) and the Hainan Island region of China (12). These changes may be closely related to shifts in population immunity and virus transmission dynamics in the post-pandemic era (13), underscoring the need for updated local epidemiological data to guide clinical practice. Furthermore, coinfections with other respiratory pathogens are common and may influence disease severity, yet detailed contemporary data on HPIV coinfection patterns in this new context remain valuable.

This study aimed to investigate the epidemiological characteristics, including detection rates, demographic and seasonal distributions, and coinfection patterns, of HPIV infections among children hospitalized with acute respiratory tract infections (ARTIs) at Shanghai Children’s Hospital from 2021 to 2024. The findings are intended to provide current evidence for optimizing the clinical diagnosis and management of HPIV-induced respiratory diseases in children.

## 2 Materials and methods

### 2.1 Research subjects

This retrospective cohort study enrolled 29,260 pediatric patients who were diagnosed with ARTIs at Shanghai Children’s Hospital between January 2021 and December 2024. All participants underwent nucleic acid testing for 13 common respiratory pathogens as part of routine clinical evaluation.

**Figure 1:**
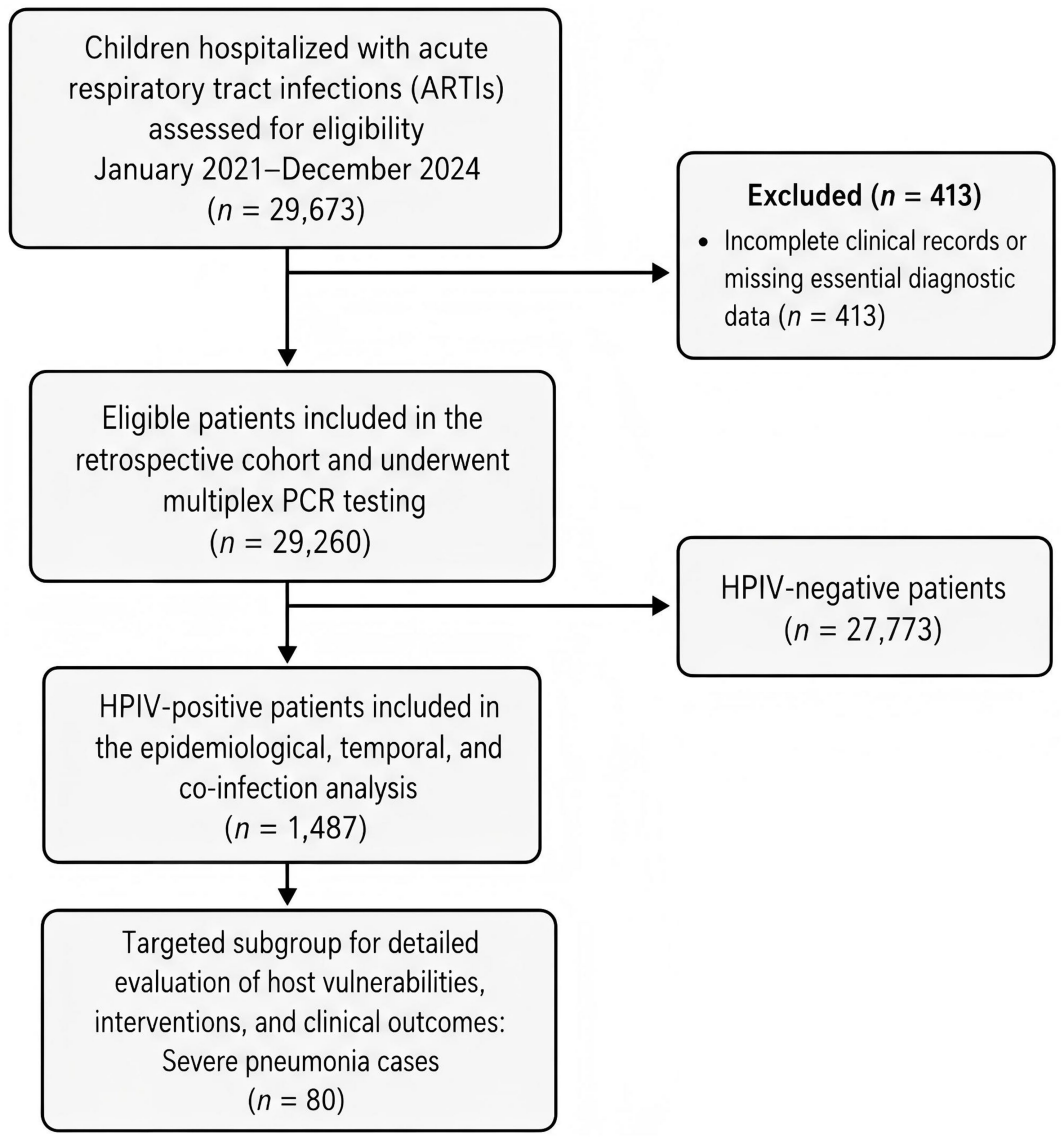
Inclusion and exclusion flowchart.

The inclusion criteria were as follows: age range of 1 month to 18 years, and a diagnosis of acute respiratory tract infections (ARTIs) according to the 8th edition of Zhu Futang’s Practical Pediatrics (14), which is explicitly defined as the acute onset of at least one respiratory symptom (e.g., cough, rhinorrhea, sore throat, tachypnea, or dyspnea) with or without fever. Severe pneumonia was defined according to the 2024 Revised Guidelines for Childhood Community-Acquired Pneumonia Management (15) as the presence of cough or difficulty breathing accompanied by at least one danger sign (such as central cyanosis, oxygen saturation < 90% on room air, or severe respiratory distress), which aligns with the widely accepted World Health Organization (WHO) criteria (16). Additionally, the term “bronchopneumonia” used in our specific clinical setting refers to lower respiratory tract infections characterized clinically by fever, cough, and localized fine crackles on auscultation, along with patchy infiltrates distributed along the bronchial tree on chest radiography, distinguishing it from lobar pneumonia and bronchiolitis. The exclusion criteria for patients were incomplete clinical records or missing essential diagnostic data (Figure 1). Furthermore, to comprehensively evaluate disease burden and host vulnerabilities, detailed clinical information was retrospectively extracted from electronic medical records for a targeted subgroup of severe pneumonia cases. This specific data extraction included underlying medical conditions (e.g., prematurity, congenital heart disease), respiratory support requirements (supplemental oxygen, mechanical ventilation), pharmacological treatments (antibiotics, systemic corticosteroids), and clinical outcomes (length of hospital stay, pediatric intensive care unit [PICU] admission, and mortality).

### 2.2 Experimental grouping

All patients were divided into four groups based on the year: 2021, 2022, 2023, and 2024; into five age groups: <1 year, 1–3 years, 4–6 years, 7–12 years, and 13–18 years; and into four seasons: March–May (spring), June–August (summer), September–November (autumn), and December–February (winter). The distribution of patients across these groups was analyzed descriptively. In comparative analyses (e.g., detection rates across years, seasons, or age groups), the Chi-square test was used. This study was approved by the Ethics Committee of the Children’s Hospital of Shanghai/Shanghai Children’s Hospital, Shanghai Jiao Tong University (Approval No: 2025R005-E01). All methods were carried out in accordance with the Declaration of Helsinki. Informed consent was waived as per ethical approval, as the research involved de-identified data and did not compromise privacy or commercial interests.

### 2.3 Sample collection

In children clinically diagnosed with acute respiratory tract infections (ARTIs), standardized throat swab samples were collected by trained healthcare personnel via sterile synthetic fiber swabs (e.g., nylon or Dacron) within 24 h of hospital admission. The collection procedure involved gently swabbing both the tonsillar fossae and the posterior pharynx under adequate lighting to ensure sample quality. Immediately after collection, swabs were immersed in 3 mL of viral transport medium (VTM) containing antimicrobial agents (e.g., gentamicin and amphotericin B) to prevent bacterial/fungal overgrowth. The samples were labeled, sealed in leak-proof biohazard bags, and transported on ice to the laboratory within 2 h of collection. Upon receipt, the samples were aliquoted to avoid repeated freeze–thaw cycles, stored at −20 °C in a dedicated medical freezer until nucleic acid extraction, and typically processed within 72 h to preserve RNA/DNA integrity. Quality control measures included documentation of collection time, visual inspection for leakage, and rejection of inadequately labeled or contaminated samples.

### 2.4 Experimental testing and methods

Multiple rounds of PCR and capillary electrophoresis were used to detect 13 viruses and atypical pathogens associated with respiratory infections. The detected viruses included HPIV, human adenovirus (HAdV), influenza A virus (FluA), influenza A virus H3N2, influenza A virus H1N1, influenza B virus (FluB), human rhinovirus (HRV), human respiratory syncytial virus (HRSV), human metapneumovirus (HMPV), human coronavirus (HCoV; referring specifically to seasonal human coronaviruses and excluding SARS-CoV-2), and human bocavirus (HBoV). Furthermore, the two atypical respiratory pathogens detected were *Mycoplasma pneumoniae* (MP) and *Chlamydia pneumoniae* (CP). Both the nucleic acid extraction kit and the multiplex PCR kit for respiratory pathogens were purchased from Ningbo Haier Genomics Technology Co., Ltd. The procedures and interpretation of the results strictly followed the kit instructions. The kit includes positive and negative controls that participate in the entire experimental process, monitoring sample extraction, nucleic acid amplification, and capillary electrophoresis. It uses endogenous internal references to monitor the quality of samples through peak detection of endogenous RNA and DNA. The Smart Lab instrument assisted by a fully automatic nucleic acid extractor extracts total nucleic acids from pathogens, whereas the Thermo Fisher ProFlex TM PCR system performs multiple reverse transcription and PCR. An ABI 3500 Dx gene analyzer was used to conduct capillary electrophoresis on the amplified products, and the size and peak height of the amplified fragments were analyzed via Gene Mapper (ABI Prism) to obtain the detection results for the corresponding pathogens. Additionally, for the subset of critically ill patients who required mechanical ventilation, lower respiratory tract specimens (tracheal secretions) were routinely collected upon intubation and subjected to standard bacterial culture to evaluate for secondary bacterial superinfections.

### 2.5 Statistical methods

Data organization was conducted via Microsoft Excel 2016, and statistical analysis was performed via SPSS 27.0 software. Categorical variables were described using frequencies and percentages. Continuous variables that did not conform to a normal distribution (such as age and length of hospital stay) were expressed as the median with interquartile range (Q1, Q3). Differences in HPIV detection rates across sexes, age groups, years, and seasons were analyzed using the χ^2^ test. For comparisons involving more than two groups, post-hoc pairwise comparisons were performed with Bonferroni correction to control for the family-wise error rate. The absolute difference in proportions (risk difference) and its 95% confidence interval (CI) were calculated for key comparisons to quantify the effect size. To assess the independent association between mixed HPIV infection and severe pneumonia while adjusting for potential confounders (age, sex, year, season), multivariable logistic regression was employed, with results presented as adjusted odds ratios (aORs) and their 95% CIs. A two-tailed significance level of *α* = 0.05 was used for all tests, and *p* < 0.05 indicated statistical significance.

## 3 Results

### 3.1 Baseline characteristics

From January 2021–December 2024, a total of 29,260 children hospitalized with ARTIs were included, ranging in age from 1 month to 18 years, with a median age of 4 years; 54.9% (16,056/29,260) were male, and 45.1% (13,204/29,260) were female, as shown in Table 1.

**Table 1:** Demographic characteristics and detection rates of human parainfluenza virus (HPIV).

Feature	Positive cases (*n*)	Total tests (*n*)	Detection rate (%)	Difference vs. ref. % (95% CI)
Sex				(Ref.: female)
Male	845	16,056	5.3	0.4 (−0.1 to 0.9)
Female	642	13,204	4.9	—
Age group				(Ref.: 13–18 years)
<1 year	345	4,658	7.4	5.7 (4.4 to 7.0)*
1–3 years	676	8,350	8.1	6.4 (5.2 to 7.6)*
4–6 years	277	7,683	3.6	1.9 (0.8 to 3.0)*
7–12 years	169	7,421	2.3	0.6 (−0.2 to 1.4)
13–18 years	20	1,148	1.7	—

CI, confidence interval; Ref., reference group. Differences in detection rates and their 95% CIs were calculated. * indicate that the 95% CI does not include zero (*p* < 0.05). Overall comparisons: sex (χ^2^ = 2.411, *p* = 0.120); age group (χ^2^ = 391.702, *p* < 0.001).

### 3.2 Demographic distribution characteristics of HPIV-positive children

From January 2021–December 2024, 1,487 cases of HPIV positivity were identified, with a positivity rate of 5.1% (1,487/29,260). The positivity rates for male and female patients were 5.3% (845/16,056) and 4.9% (642/13,204), respectively. The difference was not statistically significant (absolute difference, 0.4%; 95% CI, −0.1–0.9%; χ^2^ = 2.411, *p* = 0.120). The age range of the 1,487 HPIV-positive children was from 1 month to 18 years, with a median age of 2 years. HPIV positivity was detected in all age groups. Compared with the oldest group (13–18 years, 1.7%), the positivity rate was significantly higher in children aged 1–3 years (absolute difference, 6.4%; 95% CI, 5.2–7.6%), <1 year (absolute difference, 5.7%; 95% CI, 4.4–7.0%), 4–6 years (absolute difference, 1.9%; 95% CI, 0.8–3.0%), and 7–12 years (absolute difference, 0.6%; 95% CI, −0.2–1.4%). There was a statistically significant overall difference in positivity rates across age groups (χ^2^ = 391.702, *p* < 0.001), as detailed in Table 1.

### 3.3 Time distribution characteristics of HPIV-positive children

HPIV was detected in each year from 2021–2024. The detection rate was highest in 2021 at 7.2% (364/5,053), significantly greater than that in 2022 (absolute difference, 2.9%; 95% CI, 2.0–3.8%), 2023 (absolute difference, 2.2%; 95% CI, 1.5–2.9%), and 2024 (absolute difference, 2.8%; 95% CI, 2.0–3.6%). The overall difference across years was statistically significant (χ^2^ = 61.005, *p* < 0.001) (Table 2).

**Table 2:** Temporal distribution of human parainfluenza virus (HPIV) detection rates.

Feature	Positive cases (*n*)	Total tests (*n*)	Detection rate (%)	Difference vs. ref. % (95% CI)
Year				(Ref.: 2021)
2021	364	5,053	7.2	—
2022	168	3,893	4.3	−2.9 (−3.8 to −2.0)*
2023	525	10,591	5.0	−2.2 (−2.9 to −1.5)*
2024	430	9,723	4.4	−2.8 (−3.6 to −2.0)*
Season				(Ref.: summer)
Spring	278	7,099	3.9	−5.5 (−6.3 to −4.7)*
Summer	631	6,698	9.4	—
Autumn	351	8,202	4.3	−5.1 (−5.8 to −4.4)*
Winter	227	7,261	3.1	−6.3 (−7.1 to −5.5)*

CI, confidence interval; Ref., reference group. Differences in detection rates and their 95% CIs were calculated. * indicate that the 95% CI does not include zero (*p* < 0.05). Overall comparisons: year (χ^2^ = 61.005, *p* < 0.001); season (χ^2^ = 349.919, *p* < 0.001).

A clear seasonal pattern was observed. The detection rate peaked in summer at 9.4% (631/6,698), which was significantly higher than in spring (absolute difference, 5.5%; 95% CI, 4.7–6.3%), autumn (absolute difference, 5.1%; 95% CI, 4.4–5.8%), and winter (absolute difference, 6.3%; 95% CI, 5.5–7.1%). The overall difference across seasons was statistically significant (χ^2^ = 349.919, *p* < 0.001) (Table 2).

**Figure 2:**
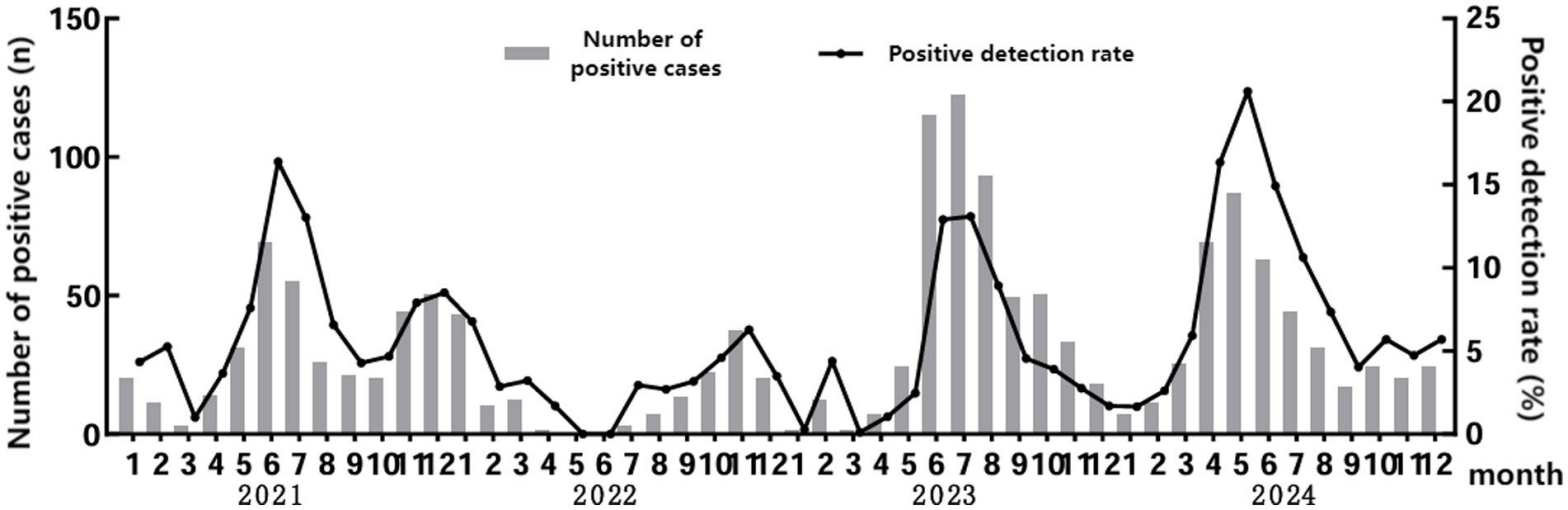
Monthly distribution of HPIV positivity rates among children in Shanghai from 2021–2024.

The monthly distribution of HPIV positivity from 2021–2024 is shown in Figure 2. HPIV was detected in every month except for May and June of 2022. In 2021, the detection rate was lowest in March (0.8%), increased to a peak of 19.0% in June, declined to 5.5% in October, and rose again to 12.1% in December. In 2022, the rate peaked at 25.6% in January, fell to zero from May to June, and increased again to 22.0% in November. In 2023, the rate increased from May, peaked at 23.2% in July, and declined steadily after September.

### 3.4 Mixed detection of HPIV and other respiratory pathogens

Among the 1,487 HPIV-positive samples, 429 were codetected with other respiratory pathogens, resulting in a mixed detection rate of 28.9% (429/1487). Among these, the dual infection rate was 24.5% (365/1487), the triple infection rate was 3.9% (58/1487), and the quadruple or higher infection rate was 0.4% (6/1487). For dual infections, the mixed detection rates for HPIV + HRV (35.2%, 151/429), MP (14.5%, 62/429), and HRSV (13.3%, 57/429) were the highest. Among the triple infections, the most common types were HPIV + MP + HRV (1.9%, 8/429), HPIV + HRV + HAdV (1.9%, 8/429), and HPIV + HBoV + HRV (1.9%, 8/429). A total of six cases of quadruple or higher infections were detected. See Table 3 for details. Among severe pneumonia patients, mixed infections primarily included HPIV + HRV (13.4%, 29/216), HPIV + MP (10.6%, 23/216), and HPIV + HRSV (4.6%, 10/216). Furthermore, among the 19 severe patients who required invasive mechanical ventilation, tracheal secretion cultures confirmed secondary bacterial co-infections in 5 cases (26.3%), which specifically included *Haemophilus influenzae* (4/5) and *Streptococcus pneumoniae* (1/5).

**Table 3:** Detection of mixed infection with HPIV and other respiratory pathogens.

Infection type	Cases (*n*)
Double infections (*n* = 365)	
HPIV + HRV	151
HPIV + MP	62
HPIV + HRSV	57
HPIV + HAdV	33
HPIV + HBoV	20
HPIV + HMPV	19
HPIV + HCoV	13
HPIV + H3N2	5
HPIV + H1N1	3
HPIV + FluB	2
Triple infections (*n* = 58)	
HPIV + MP + HRV	8
HPIV + HRV + HAdV	8
HPIV + HRV + HBoV	8
HPIV + HRV + HRSV	7
HPIV + HMPV + HRV	5
HPIV + HMPV + FluB	1
HPIV + HMPV + HRSV	1
HPIV + MP + HBoV	1
HPIV + HRSV + H3N2	1
HPIV + HRV + Cp	1
HPIV + HCoV + HBoV	1
Quadruple or higher (*n* = 6)	
HPIV + MP + HRV + HRSV	2
HPIV + MP + HRV + HAdV	1
HPIV + HRSV + HBoV + HRV + MP	1
HPIV + HRSV + HBoV + HRV + H3N2	1
HPIV + HMPV + MP + HAdV	1

HPIV: Human Parainfluenza Virus; HRV: Human Rhinovirus; MP: *Mycoplasma pneumoniae*; HRSV: Respiratory Syncytial Virus; HAdV: Human Adenovirus; HBoV: Human Bocavirus; HMPV: Human Metapneumovirus; HCoV: Human Coronavirus; FluB: Influenza B Virus; Cp: *Chlamydia pneumoniae*.

### 3.5 Clinical diagnosis of HPIV-positive children

Among the 1,487 HPIV-positive children, bronchopneumonia was the most common clinical diagnosis (66.6%, 990/1487), followed by acute bronchitis (17.1%, 254/1487) and severe pneumonia (14.5%, 216/1487). The distribution of clinical diagnoses varied significantly by age (Table 4). The proportion of bronchopneumonia cases was highest in children aged 1–3 years (48.1% of all bronchopneumonia cases). In contrast, both bronchiolitis (81.8% of all bronchiolitis cases) and severe pneumonia (23.6% of all severe pneumonia cases) were predominantly observed in children under 1 year old.

**Table 4:** Distribution of clinical diagnoses among HPIV-positive children, by age group.

Age group	Acute URI *n* (%)	Acute laryngitis *n* (%)	Acute bronchitis *n* (%)	Bronchiolitis *n* (%)	Bronchopneumonia *n* (%)	Severe pneumonia *n* (%)
<1 year	27 (18.2)	10 (35.7)	44 (17.3)	9 (81.8)	224 (22.6)	51 (23.6)
1–3 years	61 (41.2)	14 (50.0)	121 (47.6)	1 (9.1)	476 (48.1)	90 (41.7)
4–6 years	33 (22.3)	2 (7.1)	52 (20.5)	0 (0.0)	180 (18.2)	38 (17.6)
7–12 years	25 (16.9)	2 (7.1)	35 (13.8)	1 (9.1)	96 (9.7)	30 (13.9)
13–18 years	2 (1.4)	0 (0.0)	2 (0.8)	0 (0.0)	14 (1.4)	7 (3.2)
Total	148 (100)	28 (100)	254 (100)	11 (100)	990 (100)	216 (100)
χ^2^ (*p* value)	7.584 (0.108)	5.477 (0.242)	5.548 (0.236)	24.067 (<0.001)	10.075 (0.039)	10.126 (0.038)

URI, Upper Respiratory Infection. Percentages represent the distribution of each clinical diagnosis across age groups. Diseases are ordered from left to right according to increasing clinical severity.

Clinical diagnoses also differed between children with single and mixed HPIV infections (Table 5). The proportions of acute laryngitis, bronchiolitis, and acute bronchitis were numerically higher in the single-infection group, but these differences were not statistically significant (all *p* > 0.05). In contrast, the mixed-infection group was observed to have significantly higher proportions of bronchopneumonia (absolute difference, 6.6%; 95% CI, 1.4–11.8%) and severe pneumonia (absolute difference, 10.7%; 95% CI, 7.0–14.4%) compared to the single-infection group.

**Table 5:** Comparison of clinical diagnoses between children with single and mixed HPIV infections.

Diagnosis	Single infection (*n* = 1,056), *n* (%)	Mixed infection (*n* = 431), *n* (%)	Difference (mixed vs. single) % (95% CI)
Acute upper respiratory infection	105 (9.9)	43 (10.0)	0.1 (−3.0 to 3.2)
Acute laryngitis	24 (2.3)	4 (0.9)	−1.4 (−2.7 to −0.1)
Acute bronchitis	190 (18.0)	64 (14.9)	−3.1 (−7.0 to 0.8)
Bronchiolitis	8 (0.8)	3 (0.7)	−0.1 (−0.8 to 0.6)
Bronchopneumonia	684 (64.7)	306 (71.3)	6.6 (1.4 to 11.8)*
Severe pneumonia	121 (11.4)	95 (22.1)	10.7 (7.0 to 14.4)*

CI, confidence interval. The difference in percentage points and its 95% CI were calculated for the prevalence of each diagnosis in the mixed-infection group compared to the single-infection group. * indicate that the 95% CI does not include zero (*p* < 0.05). Diagnoses are ordered from top to bottom according to increasing clinical severity.

To further convey the actual clinical severity, management, and host vulnerabilities associated with HPIV infections, detailed clinical characteristics were evaluated in a subset of severe pneumonia cases (*n* = 80). We observed that 27.5% (22/80) of these severe patients had pre-existing comorbidities or a history of prematurity. The most prevalent underlying condition was congenital heart disease (12.5%, 10/80), followed by prematurity (8.8%, 7/80), with other conditions including asthma and immunodeficiency. Regarding respiratory support, 52.5% (42/80) of these severe patients required supplemental oxygen therapy, and 23.8% (19/80) progressed to require mechanical ventilation. Notably, among the critically ill subset requiring mechanical ventilation (*n* = 19), the prevalence of underlying conditions was disproportionately high at 42.1% (8/19), with structural heart anomalies being the most frequently identified risk factor (26.3%, 5/19). Due to the inherent limitations of our retrospective electronic medical records, specific data on the use of non-invasive positive pressure ventilation (NPPV) and the exact duration of oxygen therapy could not be systematically extracted. Pharmacologically, 97.5% (78/80) were treated with empirical or targeted antibiotics, and 58.8% (47/80) received systemic corticosteroids. In accordance with current pediatric antimicrobial guidelines highlighting an unfavorable risk–benefit profile, ribavirin was not utilized for HPIV management in our clinical practice. Finally, the clinical outcomes revealed a median length of hospital stay of 9 days (interquartile range: 7–15 days). Admission to the PICU was required in 35.0% (28/80) of the cases, and the overall mortality rate in this severe cohort was 2.5% (2/80); notably, these fatal cases occurred following PICU admission and are included in the PICU statistics. Furthermore, among this severe cohort, 8 patients were identified as having a single HPIV infection without any detected copathogens. These single-infection cases exhibited prominent clinical pathology, including elevated tissue damage markers (median lactate dehydrogenase [LDH] 494 U/L). Additionally, 62.5% (5/8) of these specific single-infection patients required oxygen therapy, 12.5% (1/8) required mechanical ventilation, and 37.5% (3/8) were admitted to the PICU, with no fatalities observed in this specific subset (0/8), and all (8/8) had confirmed pulmonary infiltrates on imaging.

### 3.6 Multivariable analysis of factors associated with severe pneumonia

To further assess the independent association between mixed HPIV infection and disease severity while controlling for potential confounders, a multivariate logistic regression analysis was performed, including all 1,487 HPIV-positive children. The outcome variable was the presence of severe pneumonia. The analysis adjusted for age group, sex, year of admission, and season. After adjustment, mixed HPIV infection remained independently associated with a higher risk of severe pneumonia (adjusted odds ratio [aOR], 2.45; 95% CI, 1.86–3.23; *p* < 0.001) compared to single HPIV infection (Table 6). Age was also a strong independent risk factor associated with severe pneumonia. Children under 1 year of age had significantly higher odds of severe pneumonia compared to those aged 13–18 years (aOR, 3.12; 95% CI, 1.40–6.98; *p* = 0.006).

**Table 6:** Multivariable logistic regression analysis of factors associated with severe pneumonia among HPIV-positive children.

Variable	Category	Adjusted OR (95% CI)	*p* value
Infection type	Mixed (vs. single)	2.45 (1.86–3.23)	<0.001
Age group	<1 year (vs. 13–18 yrs)	3.12 (1.40–6.98)	0.006
	1–3 years (vs. 13–18 yrs)	2.01 (0.93–4.36)	0.076
	4–6 years (vs. 13–18 yrs)	1.89 (0.85–4.20)	0.119
	7–12 years (vs. 13–18 yrs)	1.65 (0.73–3.73)	0.232
Sex	Male (vs. female)	1.11 (0.85–1.45)	0.451
Year	2022 (vs. 2021)	0.88 (0.60–1.28)	0.498
	2023 (vs. 2021)	0.94 (0.71–1.25)	0.679
	2024 (vs. 2021)	0.89 (0.66–1.20)	0.438
Season	Summer (vs. spring)	1.21 (0.85–1.71)	0.293
	Autumn (vs. spring)	1.05 (0.75–1.48)	0.772
	Winter (vs. spring)	0.92 (0.64–1.33)	0.667

OR, odds ratio; CI, confidence interval. The outcome is severe pneumonia (yes/no). The model included all variables listed.

## 4 Discussion

In this study, the overall detection rate of HPIV was 5.1%, which is consistent with the prepandemic HPIV positivity rate in the United States (5%). Notably, the positivity rate in 2021 was significantly higher than that in other years, which may be related to the following factors: (1) The impact of dynamic adjustments in SARS-CoV-2 control measures in 2021 marked a transitional period as global efforts gradually relaxed nonpharmaceutical interventions (NPIs) for SARS-CoV-2. Multiple studies have confirmed that (12, 17, 18) NPIs (such as mask wearing and social distancing) significantly reduce the spread of respiratory viruses such as HPIV. After policy adjustments, the HPIV detection rate showed a fluctuating recovery, which closely aligns with the surge in HPIV detection following the lifting of restrictions in Finland (10). (2) Viral competition and complex competitive transmission (17, 19) among respiratory viruses. Previous studies have reported that HRV detection increased markedly in 2020 while HPIV detection decreased (17). However, the ecological interplay between HPIV and other major respiratory viruses, particularly human respiratory syncytial virus (HRSV) and influenza viruses, should also be considered in the post-pandemic context. COVID-19-related non-pharmaceutical interventions substantially disrupted the transmission of multiple pediatric respiratory viruses, especially HRSV and influenza, and the resulting reduction in natural exposure has been proposed to create an “immunity gap” or “immune debt” in children (20, 21). After the relaxation of these measures, the altered timing and intensity of HRSV and influenza circulation may have reshaped the ecological niche of other respiratory viruses. Therefore, the peak of HPIV detection in our cohort in 2021, followed by a decline from 2022 to 2024, may partly reflect post-pandemic changes in viral circulation and virus–virus interactions. However, this explanation should be interpreted cautiously, as direct evidence of competitive suppression of HPIV by HRSV or influenza was not assessed in the present study. (3) Multiple studies (12, 22) have shown that the peak activity of HPIV-3, the predominant sublineage, has shifted from the traditional winter–spring season to the summer–autumn season, possibly due to gaps in population immunity and the resumption of social activities (11, 12, 23). The characteristic peak of HPIV in the summer–autumn season in this study is consistent with the monitoring data trends of Li et al. (11) in South China and Xiao et al. (12) on Hainan Island, suggesting a need to reassess previous control strategies on the basis of seasonal patterns. (4) Changes in population immunity status and long-term low-level exposure lead to a decline in children’s immunity to HPIV, which may lead to periodic outbreaks (12, 24).

In terms of population distribution, there was no statistically significant difference in the detection rate of HPIV among children with acute respiratory infections across different sexes, which is consistent with reports in the literature (25, 26). There were significant differences in the positivity rates and clinical manifestations of HPIV infection across different age groups. The study revealed that the positivity rate in the 1–3-year-old group was significantly greater than that in the other age groups, which is consistent with data from a multicenter study in the United States (27). This phenomenon may be related to the following factors: (1) Immune development characteristics: the immune systems of children in this age group have not yet fully matured, particularly their specific immunity against respiratory viruses, which is relatively weak. After HPIV infection, these patients cannot develop long-lasting immunity, leading to repeated infections (9, 28). (2) Increased exposure risk: Children aged 1–3 years begin attending daycare centers, where the group living environment greatly increases the opportunity for virus exposure (29). (3) Differences in clinical phenotypes: Data from this study show that children in the 1–3-year-old age group are more likely to develop pneumonia after infection, resulting in higher rates of medical visits for testing (30). In this study, 66.6% of HPIV infections were cases of bronchopneumonia, which is consistent with reports in the literature that 63.4% of HPIV infections progress to pneumonia (31). Among these, the positivity rate of pneumonia is highest in the 1–3 year age group, possibly due to more complete airway development at this stage, leading to more localized bronchopneumonia (31, 32). HPIV infection can cause airway inflammation and increased mucus secretion, which can severely lead to mucus plugs blocking small airways (33). In contrast, individuals in the <1 year age group, owing to their immature immune system and susceptibility to small airway obstruction, are more prone to developing bronchiolitis and severe pneumonia (34, 35). Therefore, differential monitoring and treatment strategies should be tailored for different age groups in clinical management, especially for infants with HPIV infection, which requires increased attention. Our findings also highlight a clinical phenotype divergence: while mixed infections are predominantly associated with bronchopneumonia, single HPIV infections maintain a notable association with acute laryngitis. Classically, HPIV exhibits a strong tropism for the ciliated epithelial cells of the upper respiratory tract, serving as the primary etiological agent for croup (36). However, the presence of co-pathogens may compromise local mucociliary clearance and upper airway defenses, facilitating the downward progression of the viral load and shifting the clinical manifestation from localized laryngitis to extensive lower respiratory tract involvement, such as severe pneumonia (36, 37).

In this study, the mixed detection rate of HPIV was 28.9%. Consistent with other reports (9, 27, 30, 33), mixed infections were associated with more severe clinical outcomes in our cohort. According to multicenter data from China (30, 38), 37.1% of HPIV-positive cases have mixed infections, and the risk of severe illness in children with concurrent infections has increased. A multicenter cohort study in Jordan (9, 33) further confirmed that the incidence of lower respiratory tract diseases in the mixed infection group was 1.8 times greater than that in the single infection group (*p* < 0.01). In our study, both univariate and multivariate analyses supported this association. The mixed-infection group had significantly higher proportions of bronchopneumonia and severe pneumonia. Importantly, after adjusting for age, sex, year, and season, mixed HPIV infection remained independently associated with a more than two-fold increased odds of severe pneumonia (aOR = 2.45). Mixed HPIV infections were most frequently codetected with HRV, followed by MP. Although the exact biological mechanisms underlying this observed association were not investigated in our study, previous research suggests that viral-bacterial coinfections can lead to more severe disease through complex interactions, including compromised respiratory barrier function and altered immune responses (39, 40). Specifically, co-detection with HRV facilitates respiratory epithelial barrier disruption, which, coupled with an exaggerated pro-inflammatory cytokine cascade, may accelerate airway damage (37, 39). Furthermore, coinfection with MP has been associated with hyperactive host immune responses and delayed pathogen clearance, potentially driving the progression from mild symptoms to severe or refractory pneumonia (40, 41). Notably, MP exhibits a periodic epidemic pattern with a cycle of 3–7 years, and the prevalence of MP in 2023 may partially explain the epidemiological background of the increased mixed infection rate of MP in this study. These findings highlight the clinical relevance of detecting mixed infections, as they may identify children at higher risk for severe disease.

Although our qualitative multiplex PCR panel did not provide cycle threshold values to determine viral load, clinical evidence strongly supports HPIV as a primary pathogen rather than a mere bystander. As detailed in our results, even patients with single HPIV infections (*n* = 8) within the severe cohort exhibited robust clinical pathology, including elevated markers of tissue damage (e.g., LDH), high oxygen therapy demand, mechanical ventilation requirements, and PICU admission, alongside confirmed pulmonary infiltrates. This underscores that HPIV independently drives significant lower respiratory tract damage. In mixed infections, it is highly likely that HPIV acts synergistically with copathogens to disrupt the epithelial barrier, rather than presenting a simple primary/bystander dynamic.

Importantly, our data underscored the clinical relevance of secondary bacterial infection in the most critically ill patients. Among intubated patients, tracheal secretion cultures identified bacterial co-infections, predominantly *Haemophilus influenzae* and *Streptococcus pneumoniae*. This finding is biologically plausible, as viral lower respiratory tract infection can impair epithelial integrity, mucociliary clearance, and local immune defense, thereby facilitating bacterial adherence, proliferation, and invasion of the lower airways (42). Consistent with this mechanism, previous pediatric intensive care studies have shown that bacterial co-infection is common among children requiring invasive ventilation for severe viral bronchiolitis and is associated with prolonged mechanical ventilation and longer PICU stay (43, 44). These findings support the need for systematic microbiological surveillance in intubated children with severe viral lower respiratory tract infection.

This study has the following limitations: (1) the lack of viral load data (e.g., Ct values) from the qualitative multiplex PCR prevents us from definitively differentiating the primary pathogen from colonizers or bystanders solely based on viral titers, particularly in mixed infections; (2) the retrospective design and constraints of the electronic medical record system precluded the systematic extraction of complete underlying comorbidity data for the entire cohort of 1,487 HPIV-positive patients, limiting our ability to perform fully powered statistical association testing regarding host vulnerabilities; (3) HPIV genotyping was not performed, making it difficult to analyze the epidemiological differences among different serotypes; however, recent local surveillance data in Shanghai indicated that HPIV-3 was the predominant circulating serotype during this period, which strongly aligns with and provides virological context for the distinct summer peak observed in our cohort (45); (4) the observational nature of the study limits causal inference regarding the relationship between mixed infection and disease severity; (5) SARS-CoV-2 was not included in our 13-pathogen multiplex PCR panel but was tested via separate clinical protocols, meaning we could not systematically evaluate HPIV and SARS-CoV-2 coinfections, thus the possibility of COVID-19 coinfection impacting clinical severity in this cohort cannot be definitively ruled out; and (6) the data were collected from a single center. Multicenter prospective studies are needed in the future.

## 5 Conclusion

In summary, this study delineates a significant epidemiological burden of HPIV among hospitalized children with ARTIs in Shanghai, characterized by heightened susceptibility in children under three years of age and a pronounced seasonal peak shifting to summer and autumn. Critically, we observed that mixed HPIV infections, particularly with rhinovirus and MP, were independently associated with more severe clinical disease. These findings advocate for increased clinical vigilance and targeted testing for HPIV during peak seasons, especially in young children presenting with severe respiratory symptoms. Furthermore, the detection of a mixed HPIV infection should alert clinicians to a potentially higher risk of disease progression, guiding more intensive monitoring and informing comprehensive management strategies. This evidence contributes to optimizing the diagnostic and therapeutic approach for pediatric ARTIs in the post-pandemic era.

## Data Availability

The raw data supporting the conclusions of this article will be made available by the authors, without undue reservation.

## Glossary

HPIV: Human Parainfluenza Virus
ARTIs: Acute Respiratory Tract Infections
HRV: Human Rhinovirus
HAdV: Human Adenovirus
FluA: Influenza A Virus
FluB: Influenza B Virus
HRSV: Respiratory Syncytial Virus
HMPV: Human Metapneumovirus
HCoV: Human Coronavirus
HBoV: Human Bocavirus
MP: *Mycoplasma pneumoniae*
Cp: *Chlamydia pneumoniae*
LRTI: Lower Respiratory Tract Infection
NPIs: Nonpharmaceutical Interventions
PCR: Polymerase Chain Reaction
TLRs: Toll-Like Receptors
NF-κB: Nuclear Factor Kappa–Light–Chain–Enhancer of Activated B Cells
IL-6: Interleukin-6
TNF-*α*: Tumor Necrosis Factor Alpha
Th1: T Helper 1
Th2: T Helper 2
CARDS: Community-Acquired Respiratory Distress Syndrome.
